# Covered self-expandable metal stents in benign biliary strictures caused by chronic pancreatitis: a randomized controlled trial of 6 versus 12 months of stenting

**DOI:** 10.1007/s00464-026-12919-x

**Published:** 2026-05-18

**Authors:** Outi Lindström, Marianne Udd, Juha Grönroos, Truls Hauge, Heikki Karjula, Tuomo Rantanen, Jevgeni Sumerin, Leena Kylänpää

**Affiliations:** 1 Department of Gastrointestinal Surgery, Helsinki University Hospital and University of Helsinki, Haartmaninkatu 4, PB 340, 00029 HUS Helsinki, Finland; 2 Department of Surgery, University of Turku and Abdominal Centre, Turku University Hospital, Turku, Finland; 3 Department of Gastroenterology, Oslo University Hospital and Institute of Clinical Medicine, University of Oslo, Oslo, Norway; 4 Department of Gastrointestinal Surgery, Oulu University Hospital, Oulu, Finland; 5 Institute of Clinical Medicine, University of Eastern Finland and Department of Surgery, Kuopio University Hospital, Kuopio, Finland; 6 Department of Gastrointestinal Surgery, Wellbeing Services County of South Karelia, Lappeenranta, Finland

**Keywords:** Fully covered self-expandable metal stent, Chronic pancreatitis, Benign biliary stricture, ERCP, Endoscopic therapy, Stenting time

## Abstract

**Background:**

In chronic pancreatitis (CP), a symptomatic benign biliary stricture (BBS) evolves as an adverse event in 3–30%. Treatment by ERCP with a single plastic biliary stent has been disappointing. Multiple plastic stents are more successful but make the procedure more time consuming than treatment with a fully covered self-expandable metal stent (fcSEMS). The most efficient stenting duration of BBS caused by CP is not known.

The aim of this multicenter prospective randomized study was to compare the safety and feasibility of 10 mm diameter fcSEMSs placed for 12 months versus 12 mm diameter fcSEMSs placed for 6 months in the treatment of BBS caused by CP.

**Methods:**

The patients were randomized into two groups: either 10 mm diameter fcSEMS for 12 months or 12 mm diameter fcSEMS for 6 months. After stent removal, the patients were followed up at 6 and 24 months with liver function tests and abdominal ultrasound.

**Results:**

A total of 62 consecutive patients undergoing ERCP for the treatment of BBS at six centers were enrolled. Four patients in the 10 mm group and five in the 12 mm group were excluded due to a diagnosis of pancreatic cancer, and two in the 10 mm and four in the 12 mm group were excluded because of death from other causes during the 2-year follow-up. In the 10 mm group, one stent became impacted in the bile duct necessitating hepaticojejunostomy; this patient was also excluded from the final analysis. The final sample size was 24 patients in the 10 mm group and 22 in the 12 mm group. In the 12 mm group, one stent migrated requiring re-stenting. Recurrent strictures occurred in 3 of 24 patients (13%) in the 10 mm group and in 4 of 22 patients (18%) in the 12 mm group (*p* = 0.694).

**Conclusions:**

Both types of fcSEMS are effective in the treatment of BBS secondary to CP. However, the increased risk of pancreatic cancer in these patients should be taken into consideration.

**Graphical abstract:**

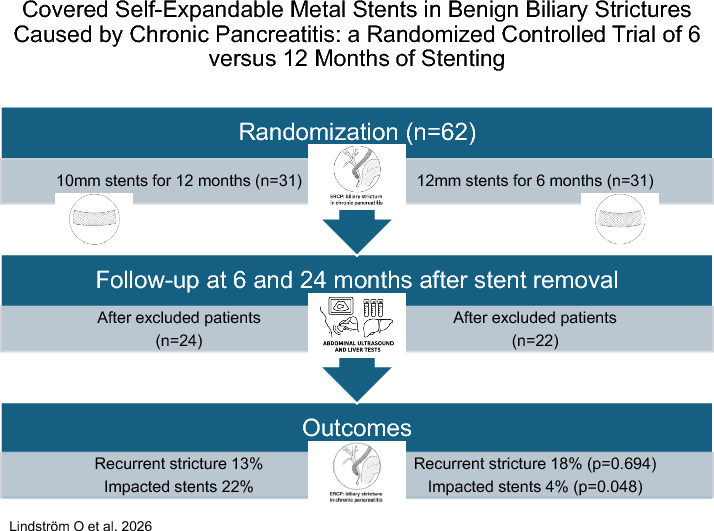

In chronic pancreatitis (CP), the recurrent inflammatory episodes lead to parenchymal fibrosis and calcification. In the pancreatic head, periductal fibrosis may cause strictures of the distal common bile duct (CBD) [1]. These strictures can be symptomatic—manifesting as abdominal pain, jaundice or cholangitis—or asymptomatic, with abnormalities detected only on imaging studies or through elevated liver function tests [2]. Some strictures may be reversible; however, in advanced CP, symptomatic benign biliary strictures (BBSs) occur in up to 30% of patients [3, 4]. CP is a well-recognized risk factor for pancreatic cancer (PC), with an almost eightfold increased risk of developing within five years of diagnosis. Moreover, in cases involving an inflammatory mass in the pancreatic head, distinguishing CP from PC can be particularly challenging [5, 6].

Endoscopy is the first-line treatment for BBSs [7]. In advanced CP, the strictures are often highly fibrotic and resistant to dilation; therefore, treatment with endoscopic retrograde cholangiopancreatography (ERCP) using a single plastic biliary stent is usually ineffective [3, 8]. Treatment with multiple plastic stents (MPSs) or fully covered self-expandable metal stents (fcSEMSs) is recommended, and the results have been comparable in terms of stricture resolution, recurrence, and adverse events. However, treatment with fcSEMSs requires fewer ERCP procedures [9].

The European Society of Gastrointestinal Endoscopy recommends the placement of MPSs or fcSEMSs for the treatment of BBSs in CP when biliary obstruction persists for at least four weeks, and in fibrotic strictures, stent therapy should be continued to achieve adequate stricture dilation [10]. The American Gastroenterological Association expert review states that ERCP with fcSEMSs placement is the preferred treatment for BBSs in CP, as it requires fewer procedures than treatment with MPSs [11]. Similarly, the American Society for Gastrointestinal Endoscopy (ASGE) guideline suggests the use of fcSEMSs over MPSs for the treatment of symptomatic BBSs in CP [12].

In previous randomized studies, stricture resolution rates in patients with CP-associated BBSs treated with MPSs or fcSEMSs have ranged from 75 to 90% during follow-up. Reported stenting durations with fcSEMSs have varied from six to 12 months, and follow-up periods have been one to two years after stent removal [13–15]. In our earlier randomized study, the stricture-free success rate with fcSEMSs was 92% at two years of follow-up [13]. To date, no randomized studies have compared different fcSEMS designs or durations of stent placement in the treatment of CP-related BBSs.

The aim of this prospective randomized study was to compare the safety and feasibility of 10 mm diameter fcSEMSs placed for 12 months versus 12 mm diameter fcSEMSs

placed for six months in the treatment of BBS caused by CP.

## Patients and methods

### Patients

A total of 62 consecutive patients with CP and BBSs, with or without acute pancreatitis, who underwent ERCP were prospectively recruited from six hospitals: Helsinki University Hospital (*n* = 46), Turku University Hospital (*n* = 3), Oulu University Hospital (*n* = 3), Kuopio University Hospital (*n* = 3), South Karelia Central Hospital (*n* = 4) in Finland, and Oslo University Hospital (*n* = 3) in Norway. Patients were randomized between September 2013 and April 2021. The study protocol was approved by the Ethics Committee of Helsinki University Hospital (HUS 175/13/03/02/13, 12.6.2013), and by the local Clinical Research Committees. All patients provided written informed consent prior to participation. The study was registered at ClinicalTrials.gov (NCT01929538).

Patient age and sex, as well as the etiology of CP, were recorded. At initial presentation, laboratory and clinical findings—including plasma bilirubin and alkaline phosphatase (ALP) levels, abdominal pain, jaundice, and cholangitis—were recorded. Pancreatic calcifications were assessed using abdominal computed tomography (CT). Patients with malignancy, known liver cirrhosis, acute or chronic hepatitis, abnormal hepatic imaging findings, or a first episode of acute pancreatitis were excluded from the study.

### ERCP procedures

All patients were prepared for and sedated during ERCP according to the hospital’s standard clinical practice. At the index ERCP, endoscopic sphincterotomy was performed, and the presence or absence of CBD stones proximal to the stricture, as well as their management, was documented. FcSEMSs could be placed and randomization performed either at the index ERCP as first-line therapy or at a subsequent ERCP following initial plastic stent placement as second-line therapy. Balloon dilatation of the CBD was performed only when considered necessary. Pancreatic stents were placed when clinically indicated.

At the randomization ERCP, patients were randomized using sealed envelopes into two groups to receive either a 10 mm or a 12 mm diameter fcSEMS in the bile duct. The stenting duration was 12 months for the 10 mm stents and six months for the 12 mm stents. The length of the fcSEMS (four or six centimeters) was selected by the endoscopist to adequately bridge the stricture. In the event of stent migration, the migrated stent was replaced with a new fcSEMS of the same type.

At the stent removal ERCP, stents were removed when technically feasible. The maximal diameter of the CBD proximal to the stricture and the length of the stricture were measured at both the initial and final ERCPs.

If a pancreatic stent had been placed, its removal or replacement was performed according to institutional practice. After each ERCP procedure, patients were hospitalized until the same evening or overnight to monitor for early adverse events. Plasma amylase levels were measured four hours after ERCP on the same day. Adverse events and their severity were defined according to established consensus criteria [16].

### Follow-up

At follow-up controls conducted at six and 24 months after stent removal, liver function tests were assessed, and abdominal ultrasound (US) was performed. These markers were selected for follow-up because they are readily available, relatively inexpensive and non-invasive. If the radiology report stated that the CBD was not dilated, a CBD diameter of 6 mm was used for analysis. Adverse events and their management, hospital admission times, surgical interventions, and any additional care required were monitored and recorded throughout the follow-up period. In addition, patients were instructed to contact the ERCP unit if they experienced symptoms or adverse events, even if these occurred after the scheduled follow-up time.

### Study endpoints

The primary endpoints of the study were long-term stricture resolution, defined by normal liver function tests and abdominal US, and recurrent stricture formation requiring additional endoscopic or surgical interventions. The secondary endpoints were stent removability at the scheduled time of the stent removal, as well as morbidity and mortality during the study period.

### Statistical analysis

In our previous study [13], sample size estimation was performed using the following assumptions: a power of 0.80 and a two-sided ⍺ of 0.05. Based on previously reported non-randomized data, the expected success rate was 90% for fcSEMS treatment compared with 60% for plastic stent treatment, resulting in a required sample size of 60 patients (30 per group). As no prior studies were available comparing different fcSEMSs, the same sample size (30 + 30 patients) was adopted for the present study.

Statistical analyses were performed using IBM SPSS Statistics (version 29; IBM Corp., Armonk, NY, USA). Categorical variables and proportions were compared using the Chi-square test or Fisher’s exact test, as appropriate, and continuous variables were compared using the Mann–Whitney U test. Data are presented as median (range) or as number of patients (%), as appropriate. A p value < 0.05 was considered statistically significant.

## Results

**Fig. 1:**
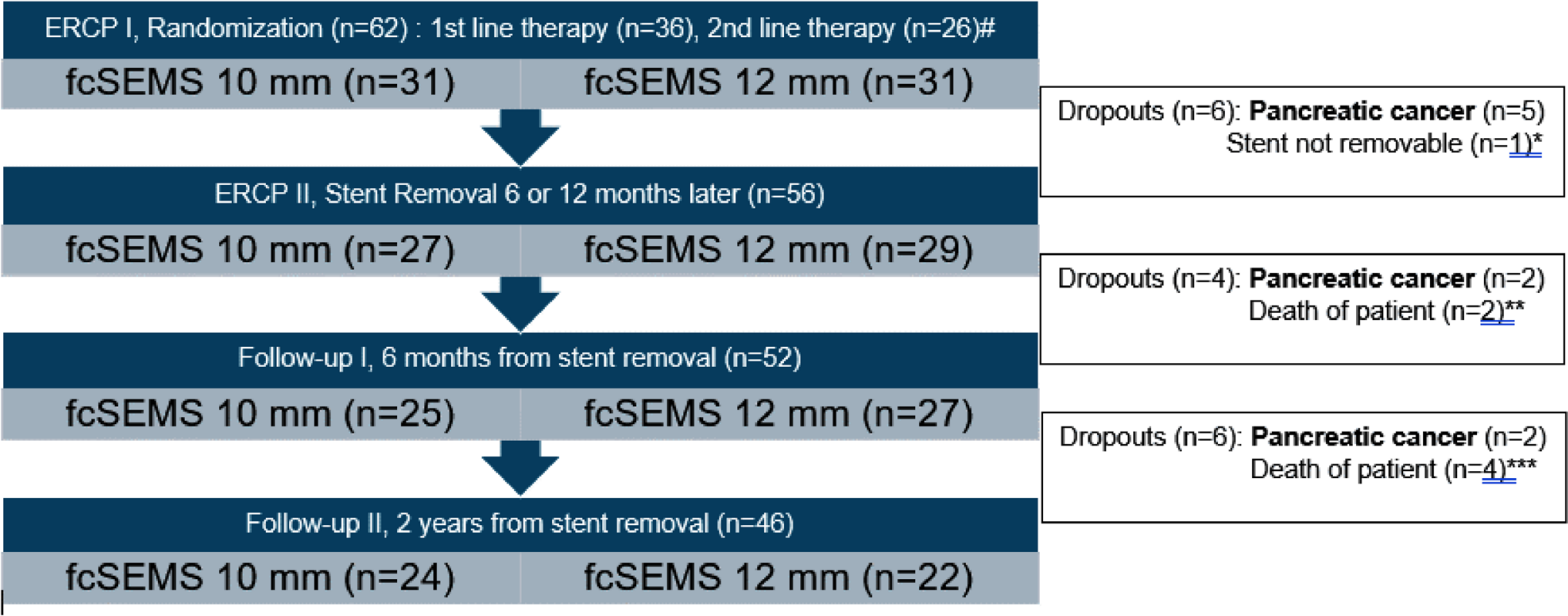
The patient flow diagram. ERCP (endoscopic retrograde cholangiopancreatography), fcSEMS (fully covered self-expandable metal stent), # 1^st^line therapy = no previous stent therapy and 2nd-line therapy = previous stent therapy with plastic stent; patients with pancreatic cancer were excluded from the analysis; *one stent got stuck and the patient was operated with hepaticojejunostomy; **one patient died of cholangitis and one of liver cirrhosis; ***one patient died of cholangitis, one of pulmonary embolism, one of COVID infection, and in one patient, the reason of death was unknown

Sixty-two patients (31 in each group) were randomized to the study. During the study period, four patients in the 10 mm group and five patients in the 12 mm group were diagnosed with PC and were excluded from the analysis. In addition, two patients in the 10 mm group and four patients in the 12 mm group died from other causes [two cases of cholangitis (one in each stent group), one case of cirrhosis, one COVID infection, one pulmonary embolism, and one unknown cause] and were therefore excluded from the final analysis. In one patient in the 10 mm group, the stent became stuck and could not be removed, necessitating surgical hepaticojejunostomy (HJ); this patient was also excluded. Consequently, the final analysis included 24 patients in the 10 mm group and 22 patients in the 12 mm group. The patient flow diagram is shown in Fig. 1.

The baseline characteristics of all randomized patients are presented in Table 1. No statistically significant differences were observed between the study groups. The median age was 59 years (37–75). 82% of the patients were male, and alcohol was the most common etiology of CP, accounting for 84% of cases. Pancreatic calcifications on CT were present in 82% of patients, and 21% had acute inflammatory features at the time of the index ERCP. Nearly 80% of patients reported abdominal pain, approximately half presented with jaundice, and one-fifth had cholangitis at the initial ERCP.

**Table 1:** Patient characteristics of all randomized patients

	Total (*n* = 62)	10 mm fcSEMS (*n* = 31)	12 mm fcSEMS (*n* = 31)	*P*
Male/female, *n* (male in %)	51/11 (82%)	26/5 (84%)	25/6 (81%)	1.000
Age (years), median (range)	59 (36–75)	56 (39–75)	61 (36–72)	0.352
Etiology of CP, *n* (%)				0.428
Alcohol	52 (84)	25 (81)	27 (87)	
Biliary	3 (5)	1 (3)	2 (6.5)	
Idiopathic	7 (11)	5 (16)	2 (6.5)	
Type of CP, *n* (%)				0.211
Chronic	49 (79)	27 (87)	22 (71)	
Acute on chronic	13 (21)	4 (13)	9 (29)	
Pancreatic calcifications, n (%)	51 (82)	27 (87)	24 (77)	0.508
Symptoms, *n* (%)				
Jaundice	31 (50)	15 (48)	16 (53)	0.800
Abdominal pain	48 (78)	26 (84)	22 (73)	0.363
Cholangitis	21 (34)	11 (36)	10 (33)	1.000

*fcSEMS* fully covered self-expandable metal stent, *CP* chronic pancreatitis, *n* number of patients

At the randomization ERCP procedure, 21 of 53 patients (40%) had or received a pancreatic stent: 11 of 27 (41%) in the 10 mm group and 10 of 26 (38%) in the 12 mm group. At the time of biliary stent removal, 17 of 53 patients (32%) had or received a pancreatic stent—10 of 27 (37%) in the 10 mm group and seven of 26 (27%) in the 12 mm group. During the follow-up period, a total of 20 patients were treated with pancreatic stents due to pain, recurrent acute pancreatitis or pancreatic fistula.

### Stricture resolution and recurrence

Plasma bilirubin and ALP levels at randomization, at stent removal, and at the six- and 24-month follow-ups; rises in these values at the six- and 24-month follow-ups; stricture lengths at randomization; and CBD diameters proximal to the stricture at randomization, at stent removal, and at the six- and 24-month follow-ups are presented in Table 2. There were no significant differences between the groups.

**Table 2:** Bilirubin and alkaline phosphatase (ALP) levels at randomization, stent removal, and six- and 24-months follow-up. Rise of bilirubin and ALP levels in six- and 24-months follow-up. Stricture length (mm) at randomization, and maximum common bile duct (CBD) diameter (mm) above the stricture at randomization, stent removal, and 6- and 24-months follow-up

Variable	All	10 mm fcSEMS	12 mm fcSEMS	p
Bilirubin *#				
Randomization	50 (3–415)(*n* = 52)	28 (3–415)(*n* = 26)	62 (3–387)(*n* = 26)	0.527
Removal	10 (2–83)(*n* = 47)	11 (2–29)(*n* = 22)	10 (4–83)(*n* = 25)	0.864
6 months	7 (3–46)(n = 43)	7 (3–46)(n = 22)	8 (3–45)(n = 21)	0.525
24 months	11 (3–261)(*n* = 40)	11 (3–261)(*n* = 21)	11 (6–108)(*n* = 19)	0.436
ALP*^				
Randomization	392 (19–1592) (*n* = 52)	303 (19–1002) (*n* = 26)	452 (68–1592) (*n* = 26)	0.151
Removal	101 (53–1339) (*n* = 50)	102 (62–735) (*n* = 25)	99 (53–1339) (*n* = 25)	0.479
6 months	86 (45–894) (*n* = 44)	91 (51–894) (*n* = 22)	85 (45–307) (*n* = 22)	0.481
24 months	102 (52–572) (*n* = 42)	110 (52–318) (*n* = 21)	87 (55–572) (*n* = 21)	0.428
Bilirubin and ALP at 6 months ¤				
Normal	29 (67)	14 (64)	15 (71)	0.747
One > RL	12 (28)	7 (32)	5 (24)	0.736
Both > RL	2 (5)	1 (5)	1 (5)	1.000
Twofold > RL	6 (14)	2 (9)	4 (19)	0.412
Bilirubin and ALP at 24 months ¤				
Normal	23 (55)	10 (48)	13 (62)	0.536
One > RL	15 (36)	10 (48)	5 (24)	0.197
Both > RL	4 (9)	1 (5)	3 (14)	0.606
Twofold > RL	7 (17)	4 (19)	3 (14)	1.000
Stricture length*&	25 (10–50)(*n* = 52)	23 (10–50)(*n* = 27)	30 (10–50)(*n* = 25)	0.850
Maximum CBD diameter*&				
Randomization	12 (8–25)(*n* = 50)	13 (9–25)(*n* = 25)	12 (8–22)(*n* = 25)	0.423
Removal	11 (6–20) (*n* = 42)	11 (6–20) (*n* = 22)	10.5 (6–15.5) (*n* = 20)	0.541
6 months	7 (3–14)(*n* = 39)	7 (4–14)(*n* = 18)	8 (3–14)(*n* = 21)	0.192
24 months	7 (4–14)(*n* = 35)	6 (4–14)(*n* = 18)	7 (5–10)(*n* = 17)	0.636

*fcSEMS* fully covered self-expandable metal stent, * median (range), *n* number of patients without: nine patients with pancreatic cancer excluded from the study population, other six dead patients and one patient with stuck stent excluded during the study period, and patients with missing or not recorded values, # < 20 umol/l, ^ 35–105 U/l, ¤ number (%), & millimeters, *RL* reference limit

At the six-month follow-up, the median bilirubin and ALP levels for all patients were within the normal range: 7 (< 20 umol/l) (3–46) and 86 (35–105 U/l) (45–894), respectively. Both liver function parameters were normal in 67% of patients, whereas 14% had values exceeding two times the upper reference limit (RL). US revealed a stricture in two patients in each stent group (4/46, 9%). Stricture recurrence requiring re-stenting occurred in two patients in each group: 2/25 (8%) in the 10 mm group and 2/27 (7%) in the 12 mm group (*p* = 1.000).

At the 24-month follow-up, the median bilirubin and ALP levels for all patients also remained within the normal range: 11 (3–261) and 102 (52–572), respectively. Both liver function values were normal in 55% of patients, while 17% had elevations exceeding two times the RL. US showed a stricture in two patients in the 12 mm group (2/46, 4%). The cumulative stricture recurrence rate requiring re-stenting was 3/24 (13%) in the 10 mm group and 4/22 (18%) in the 12 mm group, with no significant difference between groups (*p* = 0.694).

### Stent removal

**Fig. 2:**
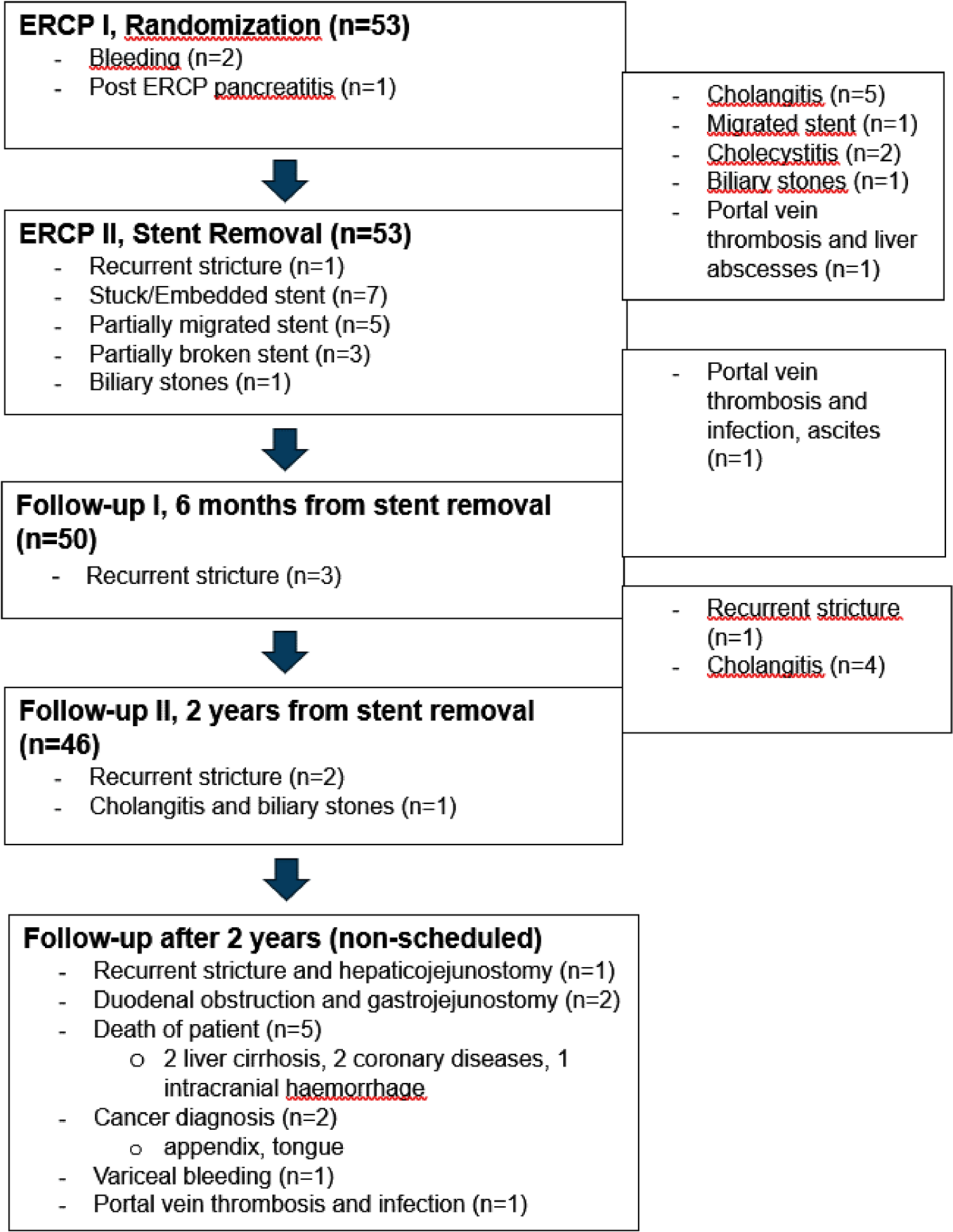
Adverse events of the procedures and patients, pancreatic cancer patients (nine patients) excluded. On the right side, adverse events were found between the controls

Adverse events related to the ERCP procedures are summarized in Fig. 2. Prior to stent removal, one 12 mm stent was found to have migrated into the bowel, and the patient was re-stented with a similar device. One 10 mm stent was so firmly impacted in the CBD that it could not be removed despite multiple attempts, and the patient ultimately required surgical intervention. At the time of stent removal, a total of six additional stents (five 10 mm and one 12 mm) were found to be stuck or embedded. Three of these were successfully removed during the same ERCP session. One patient required a second ERCP for successful stent removal, and two patients required placement of a second fcSEMS inside the original stent; at a subsequent ERCP, both stents were successfully removed. Overall, 6 of 27 (22%) 10 mm stents and 1 of 26 (4%) 12 mm stents were found to be stuck or embedded. The difference between the groups was statistically significant (*p* = 0.048).

At the time of stent removal, five stents (two 10 mm and three 12 mm) were found to be partially migrated: four within the bile duct and one into the bowel. All migrated stents were successfully removed, and none of these patients required re-stenting at that time. Among the 10 mm stents, 2 of 27 (7%) showed (partial) migration, compared with 4 of 26 (15%) 12 mm stents (three partially migrated); however, this difference was not statistically significant (*p* = 0.360). In addition, stent fracture was observed in three patients (one in the 10 mm group and two in the 12 mm group), but all fractured stents were successfully removed.

### Adverse events

In addition to stricture recurrence and issues related to stent removal or migration, other adverse events occurred in the study population and are summarized in Fig. 2. During the randomization ERCP procedures, two bleeding events were reported. One bleeding episode related to biliary sphincterotomy was managed with placement of a fcSEMS. The other bleeding event was associated with minor papillae sphincterotomy and pancreatic duct stenting performed during the same ERCP and was treated with thermal coagulation, requiring two additional duodenoscopic procedures. Over the entire study period, one case of mild post ERCP pancreatitis (PEP) and ten cases of cholangitis were documented. Three patients had biliary stones, all of which were successfully removed. During stent therapy, two patients developed acute cholecystitis and subsequently underwent cholecystectomy.

After completion of the scheduled 24-month follow-up, the median follow-up duration was 29 months (0–94). One late stricture recurrence occurred and was treated surgically with HJ. Two patients developed duodenal obstruction and underwent operative treatment with gastrojejunostomy. Five patients died from causes unrelated to CP.

### Pancreatic cancer

Nine patients were diagnosed with PC during the study period and were therefore excluded from the final analysis. In five patients, the diagnosis was established before or at the time of stent removal; in one patient at the 6-month follow-up control; and in three patients before the 24-month follow-up control. Brush cytology was performed once in six patients, three times in two patients, and four times in one patient, with all samples showing benign histology prior to the diagnosis. All patients underwent at least one imaging examination (US, CT or MRI) before the diagnosis: two patients had one (both CT scans), one had two, two had four, three had five and one had six examinations. All patients experienced pain and required multiple hospital admissions before the diagnosis was made. The final diagnosis was established or confirmed by imaging examinations in two patients, by needle biopsy in two patients, and by surgical biopsy in two patients who underwent surgery for duodenal obstruction. In two patients, the diagnosis was based on a combination of elevated tumor markers and imaging findings, and in one patient these findings were further supported by brush cytology.

## Discussion

This is the first randomized prospective study comparing two different fcSEMS for the treatment of BBSs caused by CP. The primary endpoints were adequate biliary drainage and stricture recurrence. After exclusions, the final sample comprised 24 patients in the 10 mm group and 22 patients in the 12 mm group. At the 2-year follow-up, median liver function test values were within the normal range in all patients, with no significant differences between the groups. At that time point, US revealed biliary strictures in two patients. The cumulative stricture recurrence rate was 13% in the 10 mm group and 18% in the 12 mm group (*p* = 0.694). Both types of fcSEMS were found to be effective in the treatment of CP-related BBSs.

Our finding of an overall stricture-free success rate of 85% is consistent with previous randomized studies comparing MPSs and fcSEMSs in the treatment of BBS associated with CP. In our earlier study [13], the stricture-free success rate with fcSEMSs was 92% at two-year follow-up. Corresponding success rates were 86% at 12-month follow-up in the study by Cote et al. [14] and 76% at 12-month follow-up after stent removal in the study by Ramchandani et al. [15]. In the present study, the incidence of PC was relatively high at 15%, compared with 2% in our previous study [13] and 7% in the study by Ramchandani et al. [15].

In our earlier study of BBSs in patients with CP, difficulties with removal of 10 mm fcSEMS kept in place for six months were encountered in four of 28 patients (14%) [13]. Since then, WallFlex™ biliary RX fcSEMS (Boston Scientific, USA) has been approved by the United States Food and Drug Administration for the treatment of biliary strictures due to CP with an indwell time of up to 12 months. Therefore, a 12-month stenting period was chosen for the 10 mm stents in the present study. In contrast, the 12 mm Hanarostent^R^ (Olympus EMEA) fcSEMS was a new product at the time of the study, and we were therefore reluctant to leave it in place for longer than six months. Although fcSEMSs are generally easy to remove, they may occasionally become impacted, which is a feared complication. In this study, all 12 mm stents were Hanarostents^R^ stents, whereas among the 10 mm stents, 11 were Hanarostents^R^ and 16 WallFlex™ stents. Stent removal was difficult in seven patients (13%): six 10 mm stents (four WallFlex™ and two Hanarostents^R^) and one 12 mm stent (Hanarostent^R^) became stuck. Stents with a longer indwell time, particularly the 10 mm stents, appeared more likely to become embedded, and the difference between the stent groups was statistically significant (*p* = 0.048).

Placement of an additional fcSEMS within an irretrievable stent usually resolves the problem of stent impaction, and both stents can often be removed after some weeks [17]. We successfully applied this technique in two patients; however, in one patient the approach failed, and despite multiple attempts the stent could not be retrieved. Stent removability may be compromised by tissue overgrowth at the proximal or distal end of the stent, or by loss of integrity of the stent covering, leading to tissue in-growth through the stent mesh. In CP, acute-on-chronic inflammation may trigger a hyperplastic response of the biliary duct mucosa, thereby increasing the risk of tissue overgrowth or in-growth. Currently, we limit fcSEMS placement to a maximum of 10 months, although failure of fcSEMS removal has been observed even after a stenting duration of six months [17].

In this study, the most common adverse event was cholangitis, which occurred in 10 cases. There were two bleeding episodes during the randomization ERCPs and one case of mild PEP; no perforations were observed. One might assume that the risk of PEP increases with the use of larger diameter fcSEMSs; however, only one case of PEP occurred in this series, and it was in the 12 mm fcSEMS group. This low incidence may be explained by the fact that biliary sphincterotomy was performed in all patients: in 58% during the randomization ERCP and in the remaining 42% at an earlier procedure. Our practice is to routinely perform sphincterotomy when placing fcSEMS, a strategy that has been reported to reduce the risk of PEP [18].

The risk of cystic duct occlusion and cholecystitis is known to be slightly increased with the use of fcSEMS in the CBD [19]. The ASGE guideline therefore recommends considering MPSs in patients with an intact gallbladder [12]. However, in our present series, cholecystitis developed in only 2 of 53 patients (4%). Stent migration is a well-recognized limitation of fcSEMS and has been reported to occur in up to 20–40% of cases [20]. In the current study, the overall migration rate was low at 2% (11% when partial migrations were also included). The Hanarostent^R^ features anchoring flaps designed to reduce the risk of migration, which may explain these favorable results, as most of the stents used were Hanarostents^R^ (37/53, 70%). The use of large diameter fcSEMS may pose a risk of de novo biliary strictures in a narrow CBD; however, no such strictures were observed in this series. One possible explanation is that the median CBD diameter proximal to the stricture was relatively wide—12 mm (8–25) at the time of randomization ERCP.

Overall, nine patients were diagnosed with PC and were excluded during the 2-year follow-up. All these patients had undergone at least one CT scan, and at least one brush cytology sample had been obtained. Brush cytology confirmed the diagnosis of malignancy in only one patient, in combination with imaging findings and elevated blood tumor markers. CP is a known risk factor for PC [5, 6], and our findings support this association. This study also demonstrates that diagnosing PC in a pancreas affected by chronic inflammation is challenging. Blood tumor markers were not included in our study protocol. However, in clinical practice, routine assessment of tumor markers may be useful in patients with biliary strictures associated with CP.

This study has some limitations. First, the power calculation and sample size of the patients to be randomized were based on our earlier study [13] comparing MPSs and fcSEMSs in the treatment of BBSs associated with CP, as no prior studies comparing different fcSEMSs were available. The participating hospitals differed in size, resulting in a skewed distribution of recruited patients. However, no differences in the quality of care or treatment compliance were observed between the hospitals. The number of patients decreased during follow-up because some developed PC and therefore had to be excluded. In addition, six patients died from other causes, and one patient required surgical intervention. Furthermore, some data were missing or not recorded, as shown in Table 2. The reduction in the number of study patients and the presence of missing data may represent a potential risk of bias. In addition, the use of two different stent brands (Hanarostent^R^ and Wallflex™) may have constituted a confounding factor influencing the results.

In the majority of patients, the etiology of CP was alcohol consumption, which is associated with comorbid conditions and increased mortality. Nevertheless, follow-up of the randomized patients was accomplished surprisingly well, as follow-up was incomplete in only two patients due to noncompliance. These patients, as well as others with missing data, were retained in the final analysis because their hospital records showed no evidence of biliary stricture recurrence. In addition, five patients died from other causes, and two were diagnosed with other malignancies after the scheduled follow-up period. This study demonstrates that many patients with biliary stricture caused by CP are poor candidates for surgery due to comorbidities and unhealthy lifestyles. Given the reduced life expectancy associated with CP, endoscopic treatment is generally sufficient.

Because the number of patients in the present study was limited, we believe that these findings should be confirmed in a new randomized trial with a larger study population. Such a trial could include only 10 mm fcSEMSs with varying stenting durations, for example, ranging from six to ten months.

## Conclusion

Both fcSEMS types and stenting durations were effective in the treatment of BBSs caused by CP. However, the risk of fcSEMS impaction or embedment increases with longer stenting durations, suggesting that shorter stenting periods should be considered. In addition, the increased risk of PC in this patient population should be taken into account when managing these patients.
